# The Role of Cancer-Testis Antigens as Predictive and Prognostic Markers in Non-Small Cell Lung Cancer

**DOI:** 10.1371/journal.pone.0067876

**Published:** 2013-07-23

**Authors:** Thomas John, Maud H. W. Starmans, Yao-Tseng Chen, Prudence A. Russell, Stephen A. Barnett, Shane C. White, Paul L. Mitchell, Marzena Walkiewicz, Arun Azad, Philippe Lambin, Ming-Sound Tsao, Siddhartha Deb, Nasser Altorki, Gavin Wright, Simon Knight, Paul C. Boutros, Jonathan S. Cebon

**Affiliations:** 1 Ludwig Institute for Cancer Research, Austin Health, Melbourne, Australia; 2 Medical Oncology and Thoracic Surgery departments, Austin Health, Melbourne, Australia; 3 Informatics and Biocomputing Platform, Ontario Institute for Cancer Research, Toronto, Canada; 4 Department of Radiation Oncology (Maastro), GROW-School for Oncology and Developmental Biology, Maastricht University Medical Center, Maastricht, The Netherlands; 5 Weill Cornell Medical College-New York Presbyterian Hospital, New York City, New York, United States of America; 6 St Vincent's Hospital, Melbourne, Australia; 7 University Health Network, Ontario Cancer Institute, Princess Margaret Hospital, Toronto, Canada; 8 Department of Medical Biophysics, University of Toronto, Toronto, Canada; Queen Elizabeth Hospital, Hong Kong

## Abstract

**Background:**

Cancer-Testis Antigens (CTAs) are immunogenic proteins that are poor prognostic markers in non-small cell lung cancer (NSCLC). We investigated expression of CTAs in NSCLC and their association with response to chemotherapy, genetic mutations and survival.

**Methods:**

We studied 199 patients with pathological N2 NSCLC treated with neoadjuvant chemotherapy (NAC; *n* = 94), post-operative observation (*n* = 49), adjuvant chemotherapy (*n* = 47) or unknown (*n* = 9). Immunohistochemistry for NY-ESO-1, MAGE-A and MAGE-C1 was performed. Clinicopathological features, response to neoadjuvant treatment and overall survival were correlated. DNA mutations were characterized using the Sequenom Oncocarta panel v1.0. Affymetrix data from the JBR.10 adjuvant chemotherapy study were obtained from a public repository, normalised and mapped for CTAs.

**Results:**

NY-ESO-1 was expressed in 50/199 (25%) samples. Expression of NY-ESO-1 in the NAC cohort was associated with significantly increased response rates (*P* = 0.03), but not overall survival. In the post-operative cohort, multivariate analyses identified NY-ESO-1 as an independent poor prognostic marker for those not treated with chemotherapy (HR 2.61, 95% CI 1.28–5.33; *P* = 0.008), whereas treatment with chemotherapy and expression of NY-ESO-1 was an independent predictor of improved survival (HR 0.267, 95% CI 0.07–0.980; *P* = 0.046). Similar findings for MAGE-A were seen, but did not meet statistical significance. Independent gene expression data from the JBR.10 dataset support these findings but were underpowered to demonstrate significant differences. There was no association between oncogenic mutations and CTA expression.

**Conclusions:**

NY-ESO-1 was predictive of increased response to neoadjuvant chemotherapy and benefit from adjuvant chemotherapy. Further studies investigating the relationship between these findings and immune mechanisms are warranted.

## Background

Although the overall incidence of non-small cell lung cancer (NSCLC) appears to be declining in North America, mortality has only recently plateaued [1]. More deaths are attributable to NSCLC than any other cancer [1]. As symptoms occur late, the majority of patients are diagnosed with locally advanced or metastatic disease. Management of late-stage disease has evolved to include molecular sub-typing for somatic mutations and gene rearrangements. Unfortunately the rapid translation of molecular information into the adjuvant and neoadjuvant settings has yielded conflicting and confusing results. For example, mutations in the epidermal growth factor receptor (*EGFR*) gene are clearly predictive of response to tyrosine kinase inhibitors and improved survival for metastatic patients. In locally advanced disease, however, two underpowered studies failed to reproduce this observation, and it remains unclear whether tyrosine kinase inhibitors are beneficial for early stage NSCLC [2], [3].

There is currently no consensus for the management of locally advanced (Stage IIIA) NSCLC. This remains controversial because of variations in staging techniques, conflicting trial results and idiosyncratic practices among different institutions [4]. Options include surgery followed by adjuvant chemotherapy (ACT), neoadjuvant chemotherapy (NAC) followed by surgery and definitive chemo-radiation alone or followed by surgery. To date, the only modality shown to significantly improve survival is surgical resection followed by ACT, as shown in a number of large studies [5]. Although initial studies of NAC appeared promising, enthusiasm diminished as more data from large adjuvant studies became available, leading to poor recruitment and underpowered analyses [6]. Only one study compared NAC to ACT and demonstrated equivalency in patients with early stage disease [7], although there were caveats [4].

One particular rationale for using NAC as opposed to post-operative adjuvant therapy, is that it provides valuable, potentially predictive information, based on drug sensitivity *in vivo*. In contrast current predictive markers, cannot determine whether an individual with NSCLC will respond to a given treatment [8]. This is important as response to treatment can improve resectability and may translate into improvement in survival [9]. Furthermore, the ability to predict a priori which patients will benefit makes it possible to optimize therapy and avoid unnecessary toxicity in those who are unlikely to respond.

Cancer-Testis antigens (CTAs) are molecules characterized by expression restricted to normal testis tissue but aberrant expression in a variety of cancer types, including 10–50% of NSCLC [10], [11]. Their expression is often coordinated [12] and associated with poorer clinical outcome [13] and advanced disease [14], [15]. There is some evidence that they are functional; short interfering RNA screening of a lung cancer cell line showed that several CTAs were associated with resistance to paclitaxel chemotherapy [16]. However, the CTAs identified in these screens have not been extensively studied and their presence in patient lung tumors has yet to be evaluated. The association with chemoresistance is analogous to observations in other tumor types where it has been speculated that CTAs mark more primitive, “stem-like” cells [10], [17]–[19]. Indeed the current study was initially undertaken in order to assess the relationship between these antigens and chemoresistance. It therefore came as a surprise to discover that NY-ESO-1 and to a lesser extent MAGE-A3 were associated with chemosensitivity.

While the functional role of CTAs in cancer remains unclear, the immunogenicity of these molecules has been well documented [10]. Indeed CTAs were originally identified by cloning antigens that activated cytotoxic T lymphocytes and screening patient sera for immunoreactive antibodies [20]. To date over 100 distinct CTA families have been identified [21] with approximately 30 encoded by genes located on the X-chromosome (CT-X genes). The promoter regions for all studied CT-X genes contain CpG islands and are often methylated in somatic tissues and therefore not expressed. In cancer, global demethylation of the X-chromosome including the promoter regions of CT-X genes are believed to result in the coordinated activation of multiple CTAs [10], although it is unclear whether the CTAs are bystanders or functionally participate in tumorigenesis.

To evaluate the relationship between chemosensitivity and CTA expression in NSCLC, we performed immunohistochemical studies on tumors from patients with pathological N2 (pN2) nodal involvement (at least Stage IIIA). We investigated two clinical cohorts, a pre-operative and post-operative cohort. Patients had either undergone pre-operative NAC, where we investigated the mediastinal nodal tissue prior to treatment or were treated surgically with or without adjuvant chemotherapy in the post-operative group; where we investigated the primary tumor tissue post-resection. Here we demonstrate that the CTA NY-ESO-1 is prognostic for poor clinical outcome, however also appears to be predictive for improved response and survival benefit from chemotherapy.

## Materials and Methods

### Patients and clinical specimens

Under a protocol approved by the ethics committees of Austin Health, St Vincent's Hospital and Weill Cornell Medical Centre hospitals, patients who underwent pre-operative NAC or surgery with or without ACT for pN2 disease were identified and clinical information captured retrospectively. Consent for accessing tissues and clinical records was waived on the proviso that patient details were de-identified. NAC patients were treated with at least three cycles of platinum-based chemotherapy prior to surgery. ACT patients were treated with four cycles of platinum-based chemotherapy post resection. Patients who did not receive ACT were observed without any further treatment. For the pre-operative cohort, samples obtained from mediastinal lymph node sampling prior to chemotherapy administration were used for CTA staining and DNA isolation. In the post-operative cohort, the primary surgical lung tumor specimen was used. Response to NAC was defined pathologically by down-staging of the initial lesion post-operatively. Patients were staged pre-operatively with PET scans or mediastinoscopy.

For further validation we investigated patients treated as part of the BR.10 study [22] whose tumors were subjected to molecular profiling using Affymetrix arrays. The original study randomized 482 patients to either receive adjuvant chemotherapy or observation following surgical resection, however only 133 of these samples were available for gene expression arrays [23].

### CT antigen staining

For CTA staining, 4-µm sections of formalin-fixed paraffin-embedded (FFPE) tissues were cut and stained as previously described [19]. NY-ESO-1 (E978) was obtained from the Ludwig Institute for Cancer Research and used at a concentration of 2.5 µg/mL. MAGE-A antibody (6C1) was purchased from Abcam and used at 0.3 µg/mL. MAGE-C1/CT7 antibody (CT7-33) was obtained from Santa-Cruz (cat no sc-20034) and used at 0.5 µg/mL. The detection of nuclear and/or cytoplasmic staining in any percentage of tumor cells was considered positive. Complete absence of staining was considered negative for each CTA tested. Testis tissue was used as a positive control; normal lung tissue and absence of primary antibody were used as negative controls.

### Mutational Profiling

DNA was isolated from FFPE blocks or unstained slides. For tumor blocks, an H&E slide was reviewed histologically and 1.5 mm cores were taken from the corresponding block in areas of high tumor cellularity. For slides, areas of high tumor cellularity were scraped from the slide. The tissue or core was then de-paraffinized by serial passages in xylene and alcohol, DNA isolated using DNeasy blood and tissue kits (Qiagen, Melbourne, Australia) and subjected to mutational profiling using Sequenom's MassArray platform, Oncocarta Panel v1.0, as previously described [24]. This platform interrogates 238 mutations across 19 oncogenes (http://www.sequenom.com/sites/genetic-analysis/applications/somatic-mutation-profiling.

### Microarray Analysis

CTA mRNA abundances were evaluated in the BR.10 dataset [23]. These data are publically available at the National Centre for Biotechnology Information Gene Expression Omnibus (GSE14814) and from the Director's Challenge Project [25]. These analyses were performed in R (v2.14.2). Data pre-processing was performed using RMA [26] (Affymetrix package version 1.20.0) and updated Entrez GeneID annotation [27] (hgu133ahsentrezgcdf package v14.1.0). CTA's were matched to the gene expression microarray platform via their Entrez GeneIDs. Each gene was used to median dichotomize the patient cohort. Differences in survival between the two groups were assessed with Kaplan-Meier survival curves and Cox proportional hazard ratio modeling followed by the Wald test (survival package v2.36-12) in the whole cohort, patients treated with ACT and patients not treated with ACT.

### Statistics

Differences in patient demographics for CTA positive and CTA negative patient groups were assessed with χ^2^-tests. A p-value <0.05 was considered significant. In addition, to evaluate prognostic and predictive value of CTA expression both univariate and multivariate Cox proportional hazard ratio modeling analyses were performed in R (v2.14.2, survival package v2.36-12).

## Results

### Patients

Clinicopathological data were available for 199 patients in two cohorts. The first, a pre-operative cohort, included 94 patients who underwent neoadjuvant chemotherapy followed by surgery. In the second, a post-operative cohort, 105 patients were included; 49 who had surgical resection alone, 47 who received additional adjuvant chemotherapy and 9 where adjuvant chemotherapy data was not recorded. The clinical information for each group is summarized in Table 1. Patients treated with NAC who proceeded to surgery represent a highly selected subgroup based mainly on resectability and good performance status post-chemotherapy. In the post-operative cohort, patients who did not receive chemotherapy were mainly treated prior to 2004 when adjuvant treatment was more broadly accepted.

**Table 1:** Clinicopathological features associated with CTA expression in two cohorts of patients.

Pre-operative cohort (n = 94)		Post-operative cohort (n = 105)	
Age median (range)	63 (37–82)	Age median (range)	66 (29–86)
Sex		Sex	
Male	50	Male	58
Female	44	Female	47
Stage		Stage	
IIB	3	IIIA	99
IIIA	88	IIIB	6
IIIB	3		
Histology		Histology	
Adenocarcinoma	60	Adenocarcinoma	59
Squamous	22	Squamous	30
Other	11	Other	16
NA	1		
Response		Adjuvant chemo	
Complete Response (CR)	3	No	49
Partial Response (PR)	37	Yes	47
Stable Disease (SD)	42	NA	9
Progressive Disease (PD)	11		
NA	1		
NY-ESO-1		NY-ESO-1	
−	70	−	79
+	24	+	26
MAGE-A		MAGE-A	
−	67	−	53
+	27	+	52
MAGE-C1		MAGE-C1	
−	81	−	73
+	13	+	32
Mutation status (EGFR/KRAS/TP53)		Mutation status (EGFR/KRAS/TP53)	
−	62	−	50
+	25	+	50
NA	7	NA	5

### CT Antigen expression

**Figure 1:**
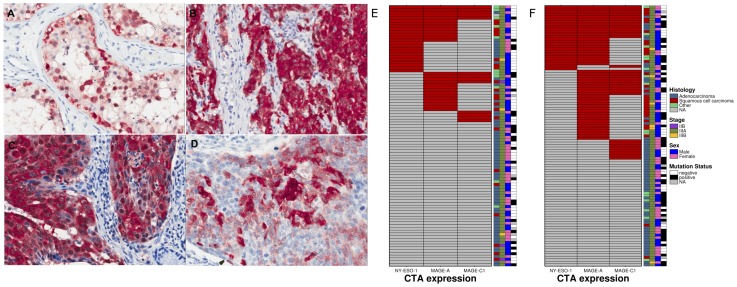
CTA expression by IHC in an individual patient tumor sample. A: Testis (positive control), B: NY-ESO-1, C: MAGE-A, D: MAGE-C1, E: Heat map detailing overlap in expression of CTAs and other clinicopathological features in the neoadjuvant cohort.

NY-ESO-1 stained similar proportions in each cohort, with 24/94 (26%) of pN2 nodes in the pre-operative cohort and 26/105 (25%) of resected lung tumors in the post-operative cohort (Figure 1). The median age of patients whose tumors stained positive for NY-ESO-1 was 62 years in the pre-operative and 66 years in the post-operative cohort, which were similar to patients with NY-ESO-1 negative tumors. More males were NY-ESO-1 positive in both cohorts with 14/24 (58%) positive in the pre-operative cohort (Pχ^2^ = 0.728) and 16/26 (62%) in the post-operative cohort (Pχ^2^ = 0.605).

MAGE-A expression was observed in only 27/94 (29%) pre-operative patients but in 52/105 (50%) in the post-operative cohort (Pχ^2^ = 0.004). MAGE-C1 stained fewer samples, with 13/94 (13%) in the NAC cohort but 32/105 (44%) in the post-operative cohort.

CTAs were expressed in both adenocarcinoma and squamous cell carcinomas (Figure 1E, 1F). However more squamous cell carcinomas were positive in both datasets, although this did not reach statistical significance in the pre-operative cohort. For NY-ESO-1 in the pre-operative cohort 8/22 (36%) squamous cell carcinomas were positive compared to 9/60 (15%) adenocarcinomas (Pχ^2^ = 0.070). Similarly in the post-operative cohort 12/30 (30%) were positive compared to 8/59 (14%; Pχ^2^ = 0.011) adenocarcinomas. These histological differences in CT antigen staining were similar for MAGE-A and MAGE-C1 and to that previously reported [13]. Furthermore expression of one CTA was often associated with expression of the others a phenomenon which has also been previously observed [13].

### Genetic mutations and CT antigen expression

We next sought to determine if CTA expression was associated with specific genetic mutations. We screened 238 somatic mutations in 19 oncogenes using Sequenom's OncoCarta panel v1.0. Lower rates of mutations in the pre-operative cohort to that expected in a Caucasian NSCLC population were observed, with only four *EGFR* (4%) mutations and nine *KRAS* (9%) mutations, all occurring in adenocarcinomas as expected. Two additional cases harbored *BRAF* mutations, while three had *TP53* mutations and one an *NRAS* mutation. The low rates of *EGFR* and *KRAS* mutations may reflect a higher rate of false-negative results due to the low tumor cell percentage in proportion to non-neoplastic cells in these lymph nodes, although areas of high tumor cellularity were specifically selected for DNA isolation. In the post-operative cohort, rates of mutations were significantly higher, and closer to those expected for late-stage NSCLC, with 16 *EGFR* mutations (15%) and 18 (17%) *KRAS* mutations. CTA expression was not associated with oncogenic mutations in adenocarcinomas (Figure 1E).

### Response to neoadjuvant chemotherapy

**Figure 2:**
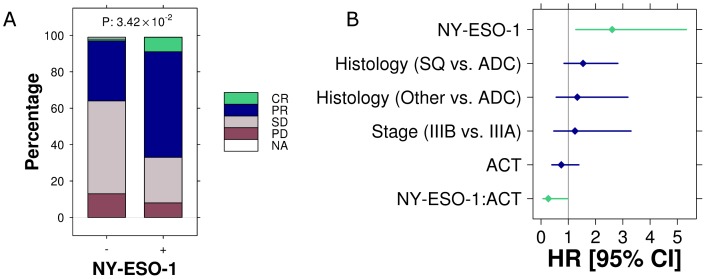
Expression of N-ESO-1 and response to neoadjuvant chemotherapy (A). CR = Complete Response, PR = Partial Response, SD = Stable Disease, PD = Progressive Disease, NA = Not Assessable. Forest plot detailing factors associated with survival in patients who were treated in the post-operative cohort. Sq = Squamous Cell, ADC = Adenocarcinoma, ACT = Adjuvant Chemotherapy (B).

Response to NAC was assessed based on pathological down staging. Of the 24 NY-ESO-1 positive tumors, two patients achieved a complete response (CR; *i.e.* no residual tumor was pathologically detectable), 14 a partial response (PR), six had stable disease (SD) and two progressed through chemotherapy (PD). In the 70 NY-ESO-1 negative patients, there was one CR, 23 PRs, 36 SD, nine PDs and one missing response (Figure 2). There was a significantly increased response rate (CR+PR) in NY-ESO-1 expressing tumors compared to NY-ESO-1 negative ones (Pχ^2^ = 0.034).

Despite improved responses to NAC, no survival differences were observed in patients expressing NY-ESO-1 or any other CTA compared to those tumors that did not. Pathological CR was associated with prolonged survival; however, for the majority of patients, the best response was a PR. It should be noted that patients that were known to have progressed through chemotherapy pre-operatively and therefore unable to proceed to surgery were not included in this study. Furthermore given the low rate of NY-ESO-1 expression, these analyses were underpowered to detect a survival difference.

### Survival following adjuvant chemotherapy

**Figure 3:**
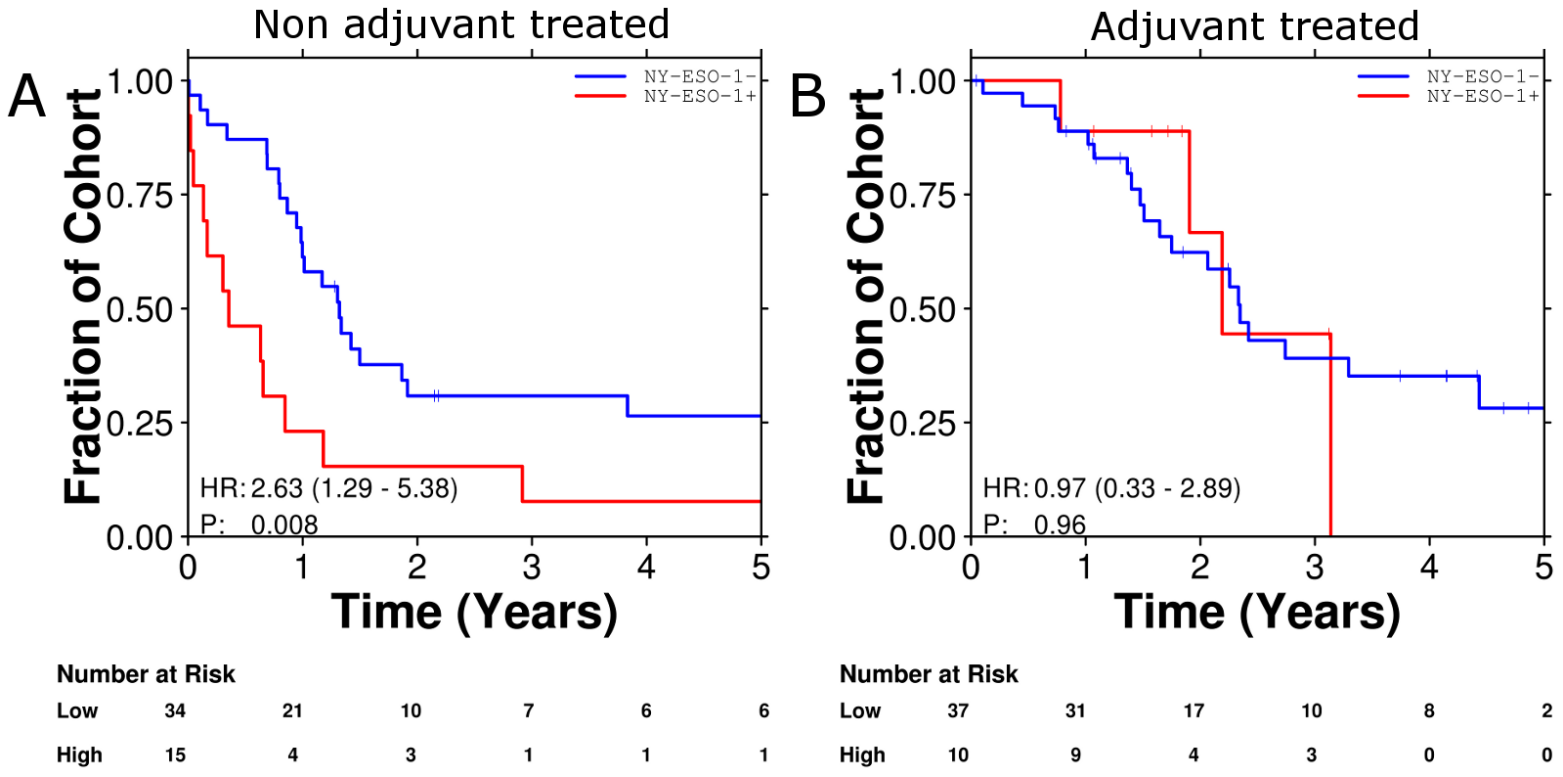
Survival curves demonstrating NY-ESO-1 to be a poor prognostic factor (A) and a predictive marker for benefit from adjuvant chemotherapy (B).

The improved responses to NAC in patients with pN2 NSCLC expressing NY-ESO-1 led us to investigate a post-operative cohort of patients with occult N2 disease, half of whom received chemotherapy in the adjuvant setting. Of 105 patients in the post-operative cohort, the use of adjuvant chemotherapy was unable to be determined in nine, leaving 96 patients for whom survival was correlated with CTA expression. Expression of MAGE-A and/or NY-ESO-1 was associated with poorer survival than MAGE-A negative or NY-ESO-1 negative tumors. MAGE-C1 expression was not associated with poorer outcome. Treatment with chemotherapy however resulted in significantly improved survival especially in NY-ESO-1 expressing tumors (Figure 3). In multivariate analysis NY-ESO-1 expression remained an independent poor prognostic factor (HR 2.61, 95% CI 1.28–5.33; p = 0.008 Wald test) whereas treatment with chemotherapy and expression of NY-ESO-1 was an independent predictor of improved survival (HR 0.267, 95% CI 0.073–0.980; p = 0.046 Wald test. Table 2).

**Table 2:** Multivariate analysis of factors associated with survival in patients treated with surgery initially.

	HR	95% CI	P (Wald test)
Stage (IIIB vs. IIIA)	1.245	0.4697–3.299	0.660
Histology (SQ vs. ADC)	1.542	0.8445–2.816	0.159
Histology (other vs. ADC)	1.331	0.5561–3.185	0.521
ACT	0.7427	0.3977–1.387	0.351
NY-ESO-1	2.609	1.278–5.329	0.008
NY-ESO-1:ACT	0.2674	0.07301–0.9795	0.046

HR = hazard ratio, ACT = adjuvant chemotherapy, SQ = squamous cell, ADC = adenocarcinoma histology.

### Survival in an independent cohort using gene expression data

**Figure 4:**
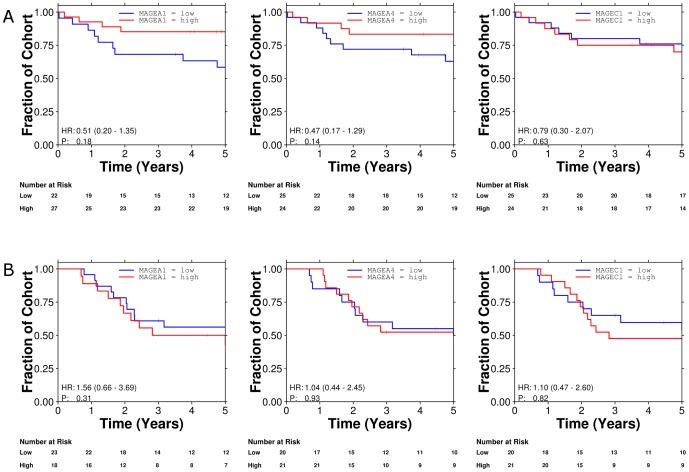
Association of MAGE-A1, MAGE-A4 and MAGE-C1 expression and survival in the BR.10 study.

We sought to validate our findings using microarray data previously published from the BR.10 adjuvant chemotherapy dataset [23]. This cohort contained Affymetrix microarray data for the mRNA abundances of 133 patients of which 71 received adjuvant chemotherapy. Unfortunately, although oligonucleotide probes representing MAGE-A1, MAGE-A4 and MAGE-C1 could be mapped to the array, NY-ESO-1 was not present after mapping to modern gene-annotations. Expression of MAGE-A1 and 4 antigens was associated with inferior survival in patients randomized to observation. However MAGE-A1 and 4 positive patients treated with chemotherapy had improved survival compared to similarly treated MAGE-A1 and 4 negative patients and survival curves similar to those seen in our post-operative cohort of patients were observed (Figure 4). However, these differences in survival did not reach the level of statistical significance. It is important to note that although survival in the overall BR.10 dataset demonstrated statistically significant differences for patients treated with adjuvant chemotherapy, in this subset of 133 patients, the trend toward improved survival remained, however was not significant. This may explain why in this underpowered cohort, similar trends were seen to those observed in our post-operative cohort although did not reach the level of statistical significance.

## Discussion

These data provide further confirmation that CTA expression in NSCLC is associated with poor overall prognosis. In particular NY-ESO-1 was associated with significantly poorer outcome in patients not treated with adjuvant chemotherapy when compared to NY-ESO-1 negative patients who were also not treated with adjuvant chemotherapy. Although similar trends were seen for MAGE-A and MAGE-C1, NY-ESO-1 remained the strongest predictor of response to chemotherapy in the neoadjuvant setting but also for benefit from adjuvant chemotherapy. These findings support NY-ESO-1 as a useful prognostic factor but also a potential predictive factor for significant benefit from adjuvant and neoadjuvant chemotherapy.

Despite the improved responses to chemotherapy in the neoadjuvant setting it was surprising that no translation from response into a survival benefit was observed for patients with NY-ESO-1+ tumors. However it should be emphasized that these analyses were underpowered to detect a survival difference. These results paralleled similar findings in patients treated with neoadjuvant chemotherapy in breast cancer. Breast tumors that were negative for estrogen receptor, progesterone receptor and HER2 gene amplification, otherwise known as “triple negative” breast cancers also strongly expressed CT-X antigens [28], [29] compared to receptor-positive tumors. Furthermore, triple negative breast cancers have been shown to be more chemosensitive to anthracycline and platinum based chemotherapy [30], [31]. Interestingly, however, although response to chemotherapy was significantly improved in triple negative tumors, survival was not impacted in these studies, except in the minority of patients who achieved a complete response. This led to the hypothesis that the poorer phenotype associated with CTA expression and triple negativity resulted in increased relapse rates, despite good initial responses [31]. These findings may explain why patients in our study whose tumors expressed NY-ESO-1 had significantly improved responses to neoadjuvant chemotherapy and yet no survival benefit when compared to patients with NY-ESO-1 negative tumors.

One caveat to our study is that the tissue investigated for our two cohorts were different, with the pre-operative cohort consisting of lymph node tissue and the post-operative cohort the primary lung tumor. There were clear differences in the number of samples that were positive for CTAs as well as for mutations between these cohorts. However in patients undergoing neoadjuvant chemotherapy, it is not possible to obtain a large portion of the primary tissue and using tissue post-chemotherapy may also be inaccurate. In our study we also tested the primary lung tumor sample after NAC for NY-ESO-1. In samples with viable tumor, NY-ESO-1 expression was not seen in the primary resected tumor if the pre-treatment lymph node was negative; however in 13 cases, despite NY-ESO-1+ lymph nodal tissue, following chemotherapy NY-ESO-1 expression was not detected in the primary resected tumor (data not shown).

As the functional role of CTAs is poorly understood, it is difficult to define a mechanism by which their expression could influence chemosensitivity. Whilst some data exist supporting *KRAS* mutations as a marker of chemoresistance and *EGFR* mutations as a marker of chemosensitivity [8], our data do not demonstrate an association between these mutations and NY-ESO-1 expression. Certainly our findings are in contradistinction to recent studies demonstrating that in cell lines, other CTAs including acrosin-binding protein (*ACRBP*) were associated with *resistance* to taxane chemotherapy [16]. However, unlike *ACRBP* which is a non-X-CTA with a specific function associated with the mitotic spindle [32], CT-X antigens evaluated in his study have no well-defined function to date. This difference likely underlies their different influences on chemosensitivity. Other studies have demonstrated that the MAGE proteins form complexes with Kap-1, a known co-repressor of *TP53* and MAGE may thus act to prevent cells from undergoing apoptosis and promote tumorigenesis. However, linking these pathways to explain the improved response to chemotherapy in breast and now lung cancer warrants further investigation.

As CTAs are known to be immunogenic, it is perhaps more plausible that the improved response and survival for patients whose tumors express NY-ESO-1 were not the result of increased cytotoxicity, but rather were mediated through immunological mechanisms. NY-ESO-1 is a potent stimulator of T-Cells and has been used as a tumor vaccine in a variety of tumor types [21], [33] Chemotherapy induced tumor cell lysis could enable antigens previously ignorant to T-Cells to be processed and presented via antigen presenting cells with resultant T-Cell stimulation. This may explain in part the increased down-staging of tumors in the short term, although one would expect a T-Cell response to result in more durable remissions. Evidence for this is supported by recent studies investigating immune checkpoint inhibitors where the impact of immune activation can be delayed such that initial disease progression may be observed followed by more durable remissions [34]. Interestingly, NY-ESO-1 specific T cell responses have been reported to increase in frequency and functionality during anti-CTLA-4 treatment, an immune checkpoint inhibitor, with resultant durable disease remissions, therefore highlighting the importance of this antigen [35]. Previous studies have not shown a correlation between the development of antibodies to tumor antigens and spontaneous remissions. However in the context of chemotherapy, immunological recognition and response in addition to chemotherapy induced depletion of regulatory T-Cells could explain the improved response rates we have observed and the longer-term survival advantage in the adjuvant setting. An alternative to these hypotheses is that the survival advantage seen in the adjuvant setting may not represent improved tumor responses but poorer survival in the absence of chemotherapy. Further studies are warranted to better explain our findings.

Recent clinical trials investigating immune checkpoint inhibitors in NSCLC have demonstrated significant promise particularly in squamous cell carcinomas [36], [37]. A study investigating a monoclonal antibody directed against CTLA-4, a molecule that inhibits T-Cell activation, demonstrated an overall benefit in prolonging immune related progression free survival. Although tumors of all different histological types appeared to benefit, a trend towards improved benefit was seen in patients with squamous cell histology, a subgroup that we have also observed to highly express CTAs [36]. These data have resulted in the launch of a randomized phase III study comparing the addition of ipilimumab to chemotherapy in patients with squamous cell histology. Similarly an inhibitor of programmed death-1 (PD-1), a molecule also involved in T-cell inhibition, has recently demonstrated efficacy in a Phase I study [37]. Significantly more responses were observed in patients with squamous cell carcinoma when compared to non-squamous tumor types. Although we are currently unable to demonstrate that CTAs play a role in these early studies, the strong association of these immunogenic molecules with the same histology to benefit from immunoactivation warrants further investigation.

These data demonstrate that expression of the CTA NY-ESO-1 is associated with increased downstaging with chemotherapy given in the neoadjuvant setting and significant survival benefit with chemotherapy given in the adjuvant setting. Our data therefore support NY-ESO-1 as both a (poor) prognostic marker and also a predictive marker. As the assay is straightforward and easy to interpret, NY-ESO-1 could easily be studied prospectively as a marker. Perhaps more importantly, the immunogenicity of this molecule may be a rationale for future studies using NY-ESO-1 as a therapeutic target, thereby better personalizing treatment towards those most likely to derive benefit.
